# The End of a Fortune

**Published:** 1881-11

**Authors:** 


### THE END OF A FORTUNE.

Perhaps there are fewer more bitter disappointments in life than the failure to realize happiness after the intense struggle for wealth and the accumulation of a fortune. Novelists and dramatists have expended skill and art in portraying the bitterness and the woe which men feel when fighting in vain to obtain wealth ; when they have almost laid their hands upon it and it eludes their grasp ; when they endure privation and hunger, the dangers of the deep and the desert, the perils of robbers and of the fire, the dishonesty of trusted clerks or partners.

The loss of property already acquired, when luxuries have become daily necessities, and those easements and conveniences which to the poor are as unattainable as royalty itself, become the habit of life, is a tragedy, and a subject for tragic treatment.

But it may be doubted if either the unavailing strife and sweat and toil for fortune, or the despair of having lost one, are more painful than the winning of one only to discover that it has brought ghastly and heart-breaking evils in its train.

What is more bleak or more barren than the outlook of one whose whole life has been devoted to the rearing of a princely fortune, and to feel that on his death it will fall to pieces; that his own children, flesh of his flesh and bone of his bone, are eagerly waiting, counting the hours and minutes as in a sick room, for him to step aside, that they may dissipate in luxury, idleness, vice or frivolity the wealth he with such pains and toil has heaped together. Even while alive he is often forced to see that his money has made them useless and indolent, or extravagant, quarrelsome and unfilial. They resent even the restraints of his advice, and assume their title to as large a share of his income as their wants rather than his wishes prescribe.

---

A WORTHY deacon in a town not far away gave notice at a prayer meeting the other night of a church meeting that was to be held immediately after, and unconsciously added : "There is no objection to the female brethren remaining."

